# Mechanism of stroke in the setting of postcardiotomy venoarterial extracorporeal membrane oxygenation support

**DOI:** 10.1016/j.xjon.2022.04.002

**Published:** 2022-04-13

**Authors:** Matan Grunfeld, Masashi Kai, Suguru Ohira

**Affiliations:** a Division of Cardiothoracic Surgery, Department of Surgery, Westchester Medical Center, New York Medical College, Valhalla, NY; b School of Medicine, New York Medical College, Valhalla, NY

To the Editor:

**Figure:**
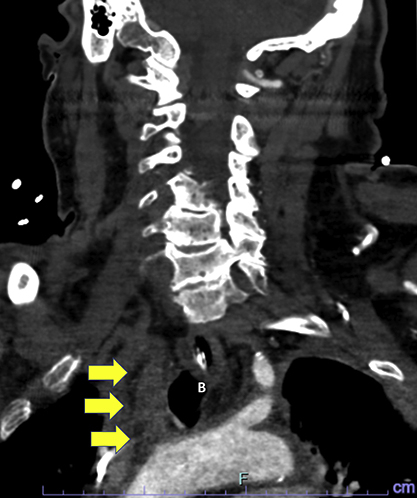

The authors reported no conflicts of interest.

The *Journal* policy requires editors and reviewers to disclose conflicts of interest and to decline handling or reviewing manuscripts for which they may have a conflict of interest. The editors and reviewers of this article have no conflicts of interest.

**Figure 1:**
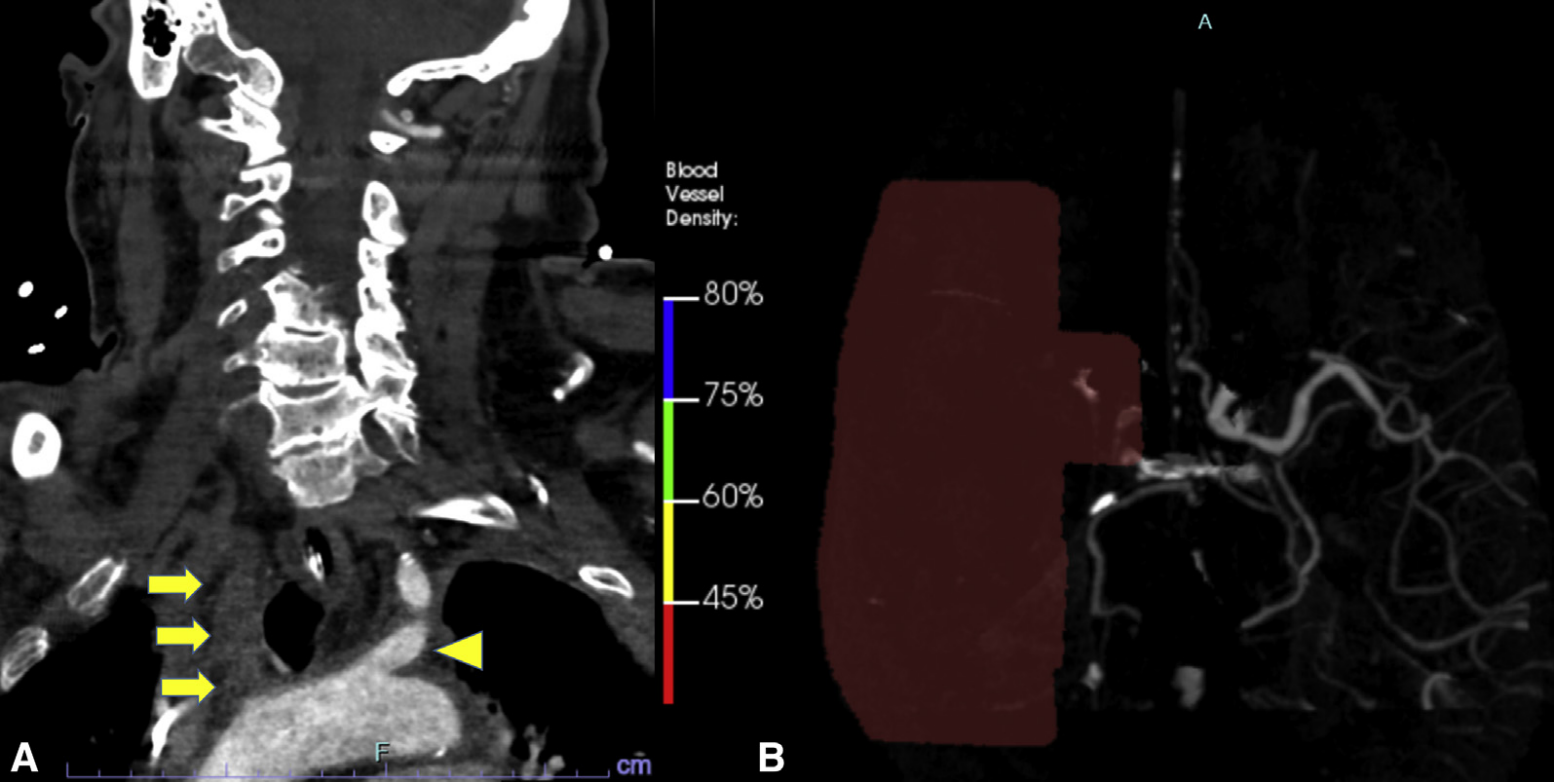
A male patient required venoarterial extracorporeal membrane oxygenation (VA-ECMO) via the right axillary artery due to postcardiotomy shock after ischemic ventricular septal defect repair. On postoperative day 2, with recovery of left ventricular function, the patient suddenly developed left hemiparesis with a dropped VA-ECMO flow. Emergent computed tomography (A) showed occlusion of the innominate artery (*arrows*) to the right common carotid artery. A brain perfusion scan showed a complete occlusion of the right middle cerebral artery system (*red area* in B). An *arrowhead* shows the left subclavian artery.

With great interest we read the study by Schaefer and colleauges,^1^ who conducted a detailed analysis of the outcomes of postcardiotomy venoarterial extracorporeal membrane oxygenation (VA-ECMO) support focusing on stroke and cannulation-related complications. The stroke rate of right axillary (RAX) VA-ECMO was greater than that of femoral cannulation. In both axillary and femoral VA-ECMO, the right hemisphere was the most common stroke location (64.5% in RAX and 50% in femoral). This stroke laterality trend in RAX cannulation was similar in our experiences.^2^^,^^3^ There are several potential embolic sources in the setting of postcardiotomy VA-ECMO support: VA-ECMO–related (circuit, tubing, or cannula), intracardiac, or arterial/aortic (atherosclerosis, suture line, or intraoperative cannulation site).^3^ Another possible mechanism of stroke in RAX VA-ECMO may be the “mixing point” between VA-ECMO flow and blood flow ejected from the heart, which can be an issue when heart function is recovered together with lower VA-ECMO flow. We experienced an occlusion of the innominate artery to the right common carotid artery after recovery of heart function in a patient postcardiotomy using RAX VA-ECMO (Figure 1).

In addition to VA-ECMO itself, the type of surgery performed is also important when discussing the cause of stroke; left-side valve surgery using prosthesis or patients with reduced ejection fraction would have a greater chance of developing an intracardiac embolic source.^3^^,^^4^ It would have been informative if the authors could have included the type of surgery in Table E6 of their article. We agree with their conclusion that surgeons understand that each configuration has advantages and disadvantages over the other, depending on the scenario of the individual.
